# Effectiveness of antibiotic-loaded bone cement in total joint arthroplasty at a tertiary medical center: A retrospective cohort study

**DOI:** 10.1016/j.jsps.2023.101739

**Published:** 2023-08-09

**Authors:** Abdullah M. Alhammad, Thamer A. Almangour, Imtinan Almasoudi, Wesal Alalayet, Mansour Almuqbil, Yazed S. Alsowaida, Naif H Alotaibi

**Affiliations:** a Department of Clinical Pharmacy, College of Pharmacy, King Saud University, Riyadh, Saudi Arabia; b Clinical Pharmacy Department, King Saud Medical City, Ministry of Health, Riyadh, Saudi Arabia; c Pharmaceutical Care Department, King Saud University Medical City, Riyadh, Saudi Arabia; d Department of Clinical Pharmacy, College of Pharmacy, Hail University, P.O. Box 6166, Hail 81442, Saudi Arabia; e Division of Infectious Diseases, Department of Internal Medicine, College of Medicine, King Saud University, PO Box 7805, Riyadh 11472, Saudi Arabia

**Keywords:** Total knee arthroplasty, Revision arthroplasty, Antibiotic-loaded bone cement

## Abstract

**Background:**

Data regarding the effectiveness of antibiotic-loaded bone cement (ALBC) in preventing prosthetic joint infections (PJI) after total joint arthroplasty (TJA) is inconsistent. The objective of this study was to evaluate if the routine use of ALBC influenced the risk of revision surgery due to PJI.

**Methods:**

This is a retrospective cohort study performed between January 2018 and September 2020. Adult patients aged ≥ 18 years who underwent TJA (knee or hip) and received either ALBC or plain cement (PC) were included. The outcome of this study was the rate of revision due to PJI. Multivariate analysis using logistic regression was used to identify factors that may be associated with increased risk of PJI, using STATA 15.1 (StataCorp LP, College Station, Texas, USA).

**Results:**

A total of 844 patients were screened and 319 patients were included. There were 247 patients in ALBC group and 72 patients in the PC group. Only vancomycin powder was used in all ALBC cases, with a 2 g dose in 50% of the cases (dose ranged between 1 g and 8 g). The status of the prosthetic joint was assessed and recorded up to 2 years of the TJA. Overall, the difference in the rates of PJI between the two groups after primary arthroplasty was not statistically significant (5.6% vs 1.4%; p = 0.173; OR, 4.2; 95% CI, 0.5–33).

**Conclusion:**

ALBC was not associated with a reduction in PJI rates after primary TJA. More research is needed to further evaluate the effectiveness of ALBC in preventing PJI.

## 1 Background

Total Joint arthroplasty (TJA) is among the major and most successful orthopedic surgeries (Martínez-Moreno et al., 2017). It is indicated when there is impaired joint function that should be replaced with prosthesis (Merola and Affatato 2019). Total hip arthroplasty (THA) and total knee arthroplasty (TKA) are the most replaced joints. The incidence of TKA tends to increase worldwide and is expected to reach 5 million degenerative knee joint replacement procedures by 2030 (Leta et al., 2021).

Around 10% of the indications for TJA are revisions replacement (Wengler et al., 2014). Periprosthetic joint infection (PJI) occurring after the TJA is a main sequela for patients and a burden on healthcare systems (Kurtz et al., 2008, Kurtz et al., 2010). It may increase the length of hospital stay, readmission rate, and lead to poor quality of life. Antibiotic-loaded bone cement (ALBC) has been suggested to decrease the risk PJI. The role of such technique is to immediately release the antibiotics at the action site with very little systemic exposure which may not be achieved by systemic antibiotic due to poor blood supply (Martínez-Moreno et al., 2017). The selection of the antimicrobial agent is still controversial and no agent has been shown to be superior. Yet, vancomycin is among the most commonly studied (Bistolfi et al., 2011, Martínez-Moreno et al., 2017). Vancomycin satisfies the properties needed to be mixed into bone cement including stability at high temperature, availability in ready-mixed solid dosage form, being a water soluble, active against the most common causative pathogens, and effective at low doses (Martínez-Moreno et al., 2017).

ALBC received the US Food and drug authority (FDA) approval for revision surgery of infected arthroplasty but not as prophylaxis in primary arthroplasty (Leta et al., 2021). It has been widely used as adjuvant therapy for patients with PJI to reduce the reinfection rate as well as in non-infected revision which is known to be associated with higher risk of infection (Carlsson et al., 1978, Hanssen, 2004, Jämsen et al., 2009); nevertheless, it has also been a routine practice in many countries to be used, although more debatable, as a prophylaxis against infection in primary arthroplasty (Hinarejos et al., 2015). The proposed benefit of using ALBC is to reduce the risk of revision surgery due to PJI and consequently the cost of care without notable side effects. However, unintended negative consequences are concerning including the adverse consequences on the mechanical and structural properties of the cement, antimicrobial resistance, allergy, and cost (Jiranek et al., 2006, Dunne et al., 2007). Despite that these concerns were not clinically validated in the literature, the benefit of using such technique should be validated to justify its use. Although ALBC has been widely accepted in revision surgeries due to PJI (Osmon et al., 2013), the use of ALBC and its potential benefit to decrease PJI risk after primary arthroplasty is controversial. The current data are insufficient, and the findings from previous studies are still inconsistent. No study has yet been published in Saudi Arabia describing the use of ALBC and its impact on the rate of PJI. Hence, the aim of this study is to evaluate if the routine use of ALBC influenced the risk of revision surgery due to PJI with specific emphases on prophylactic use in primary arthroplasty.

## 2 Methods

### 2.1 Setting and study design

This is a retrospective cohort study of patients who underwent primary TJA or revision arthroplasty including either the knee or hip with either ALBC or plain cement (PC) during the period between January 1, 2018 to September 30, 2020 at King Saud University Medical City, Riyadh, Saudi Arabia.. Patients were evaluated for potential early and late PJI. Following the surgery, participants were evaluated by their surgeons at two weeks, three months, six months, twelve months and annually thereafter. The main outcome of this study was the rate of revision due to PJI. The exposure of interest was ALBC. To be considered an infection, there should be a clear report by the treating surgeon. Diagnosis was made according to the clinical practice guideline by the Infectious Diseases Society of America (Osmon et al., 2013). This study was approved by the Institutional Review Boards (IRB 017E).

### 2.2 Participants

Adults, 18 years or older, who underwent full-cemented TJA or revision arthroplasty of the knee or hip and received either ALBC or PC due to any reason leading to the TJA were included in this study. Patients with a history of joint infection (primary arthroplasty group), allergy to the antibiotics used in the cement, underwent arthroplasties that were not fully cemented, who underwent other surgery, or who lost to follow-up were excluded. Patients who received perioperative prophylaxis except vancomycin were included.

### 2.3 Data collection

Data were collected from the electronic medical records. Demographics and baseline characteristics including gender, age, weight, body mass index (BMI), comorbid conditions, surgery type, cement type, antimicrobial dose used in cement, and pathogens causing the infection were collected. Status of the prosthetic joint was assessed and recorded up to 2 years of the TJA.

### 2.4 Definitions

Primary arthroplasty is referred to the first knee or hip prosthesis regardless of the reason for arthroplasty, while revision arthroplasty is defined as the follow-up surgery in previously replaced joint which is known to be associated with higher risk of infection. Early PJI is the infection that occurs within 1–3 months of TJA, while late PJI occurred more than three months and up to two years after TJA (Matthews et al., 2009).

### 2.5 Statistical analysis

Continuous data were presented as mean ± standard deviation (SD) if normally distributed and compared using independent *t*-test or median and interquartile range (IQR) if not normally distributed and compared using Wilcoxon rank-sum test. Nominal data were presented as counts and percentages and compared using Chi-square test. In this analysis, p values ≤ 0.05 were considered significant. Multivariate analysis using logistic regression was performed to identify risk factors for PJI. STATA 15.1 (StataCorp LP, College Station, Texas, USA) was used to conduct the statistical analysis.

## 3 Results

In this study, we screened 844 patients, among which, 319 met the inclusion criteria. Among the excluded patients, 203 underwent different surgeries and 322 were excluded due to lost to follow-up. Two hundred forty-seven patients and 72 patients were included in ALBC and PC, respectively. The average age was 65 ± 15 years and about one third were male (n = 115; 36%). Comorbidities included diabetes (n = 187; 59%), cardiovascular disease (n = 177; 55%) and renal disease (n = 12; 4%). Among patients who met the inclusion criteria, 280 and 39 patients underwent TKA and THA, respectively. Most included patients underwent primary arthroplasty (n = 269; 84%). There were higher rates of diabetes and cardiovascular diseases in PC group. Further, higher rate of patients underwent TKA in PC group versus ALBC group. Otherwise, the two groups were comparable in baseline characteristics. More details about baseline characteristics are listed in Table 1.

**Table 1:** Demographics and baseline characteristics of included patients*.

Characteristic	ALBC (n = 247)	PC (n = 72)	p value
Male, n (%)	87 (35)	28 (39)	0.325
Age in years (mean ± SD)	64 ± 15	67 ± 9	0.138
Weight in kg (mean ± SD)	82 ± 22	79 ± 12	0.655
BMI in kg/m^2^ (mean ± SD)	34 ± 9	32 ± 5	0.554
Comorbidities			
Diabetes, n (%)	127 (51)	60 (83)	< 0.001
Cardiovascular, n (%)	120 (48)	57 (79)	< 0.001
AKI, n (%)	4 (2)	1 (1)	0.890
CKD, n (%)	6 (3)	1 (1)	0.182
Joint replaced			
TKR, n (%)	208 (84)	72 (1 0 0)	< 0.001
THR, n (%)	39 (16)	None	
Type of surgery			
Primary arthroplasty, n (%)	197 (80)	72 (1 0 0)	< 0.001
Revision arthroplasty, n (%)	50 (20)	None	

Abbreviations: ALBC: antibiotic-loaded bone cement; AKI: acute kidney injury; BMI: body mass index; CKD: chronic kidney disease; PC: plain cement; TKR: total knee replacement; THR: total hip replacement.

*The χ2 test was used to compare categorical variables whereas the independent *t* test was used to compare continuous variables.

Vancomycin was the only antibiotic used in cement and the dose ranged from 1 to 8 g. The median (IQR) was 2 (1) grams. One patient received 8 g which was given to treat PJI. Specific doses are listed in Table 2**.**

**Table 2:** Vancomycin dosing in antibiotic-loaded bone cement (n = 247).

Vancomycin dose in gram/40 g of cement	Antibiotic-loaded bone cement, n (%)
1	91 (37)
1.5	7 (3)
2	137 (55)
3	4 (2)
4	7 (3)
8	1 (1)

Among all patients who underwent primary arthroplasty, 12 (4.5%) had revision due to PJI within 2 years of TJA, among which, 3 had THA and 9 had TKA. The etiologic microorganisms causing PJI were identified and reported in 8 cases: *Staphylococcus epidermidis* (3 cases), *Enterococcus faecalis*, *Staphylococcus haemolyticus*, *Streptococcus mutans*, *Bacillus licheniformis*, and *Escherichia coli,* one case each. There was no significant difference in the revision rate due to PJI in patients who underwent primary arthroplasty and received ALBC versus PC (5.6% vs 1.4%; p = 0.173; OR, 4.2; 95% CI, 0.5–33). When adjusted for age, gender, diabetes, BMI, renal diseases, cardiovascular diseases, and type of surgery in multivariate analysis using logistic regression model, receipt of ALBC did not decrease the rate PJI (adjusted OR, 5.1; 95% CI, 0.6–43) and no factor was associated with increased risk of PJI.

Among 50 patients who received revision arthroplasty, all received ALBC and 2 (4%) had PJI, both had TKA. The etiologic microorganisms causing PJI were identified and reported in one case which was *Staphylococcus haemolyticus*. More details about the outcome are listed in Table 3**.**

**Table 3:** Outcomes in patients who received ALBC and PC for primary and revision arthroplasty.

Outcome^a^	ALBC (%)	PC (%)	p value	Odds Ratio (95% CI)	Adjusted Odds Ratio^b^ (95% CI)
Primary arthroplasty (n = 269)^c^					
Periprosthetic Joint Infection	11/197 (5.6)	1/72 (1.4)	0.173	4.2 (0.5–33)	5.1 (0.6–43)
Early	6 (3.0)	1 (1.4)			
Late	5 (2.5)	None			
Revision arthroplasty (n = 50)^d^					
Periprosthetic Joint Infection	2/50 (4)	None			
Early	2 (4)	None			
Late	None	None			

Abbreviations: ALBC: antibiotic-loaded cement; PC: plain cement.

^a^Data are presented as n (%); the χ2 test was used to compare categorical variables.

^b^Adjusted for age, gender, diabetes, BMI, renal diseases, cardiovascular diseases, and type of surgery.

^c^Among 12 prosthetic joint infection cases, 9/280 (3.2%) had total knee arthroplasty and 3/39 (7.7%) had total hip arthroplasty. The single prosthetic joint infection case in plain cement group had total knee arthroplasty.

^d^Both cases with prosthetic joint infection had total knee arthroplasty.

## 4 Discussion

Our study adds to the ongoing controversy in the literature on whether ALBC is effective in preventing PJI in patients underwent primary arthroplasty. Our study main outcome was that ALBC was not associated with a reduction in PJI rates after primary knee or hip arthroplasty compared to PC in our institution.

The practice of using ALBC in primary TJA varies across different countries. Unlike in some countries such as Norway, Sweden, and United Kingdom where the use of ALBC is more common, its use is not a routine practice in the United States; however, it has been increasingly prevalent (Robertsson et al., 2001, Engesaeter et al., 2003, Malik et al., 2005, Jiranek et al., 2006, Bendich et al., 2020). The status in Saudi Arabia is unclear in the literature; nevertheless, three quarters of our study population underwent ALBC for their primary TJA. The reason for using PC is unknown. In clinical practice, however, PC could be used where there are specific contraindications of using ALBC.

Data in the last decade from *meta*-analyses (Wang et al., 2013, Zhou et al., 2015), prospective randomized trial (Hinarejos et al., 2013), retrospective observational studies from institutional or national-based registries (Bohm et al., 2014, Qadir et al., 2014, Wang et al., 2015, Jameson et al., 2019, Bendich et al., 2020, Leong et al., 2020), showed conflicting results on the effectiveness of ALBC at reducing PJI rates in primary arthroplasty. While some studies showed a reduced risk of PJI (Wang et al., 2013, Jameson et al., 2019, Bendich et al., 2020, Leong et al., 2020), others showed no difference when ALBC was compared to PC in primary arthroplasty (Hinarejos et al., 2013, Bohm et al., 2014, Qadir et al., 2014, Wang et al., 2015, Zhou et al., 2015).

The selection of vancomycin was based on several reasons. Microbiologically, it has activity against Gram-positive organisms specially *Staphylococcus aureus* and *Staphylococcus epidermidis*, the most common etiologic pathogens for PJI with low rate of resistance (Pulido et al., 2008, Li et al., 2018). Physically and chemically, it has good thermal stability and water solubility (Hinarejos et al., 2015). Pharmacologically, it has low potential for allergic reaction and systemic toxicity (Springer et al., 2004, Hsieh et al., 2006). It has good biological activity in bone cement and clinical outcomes in previous studies (Martínez-Moreno et al., 2017). It is also readily available in our institution with reasonable cost.

The retrospective nature of the design is one of the main limitations of our study which may lead to selection bias, inclusion bias, and data incompleteness. In addition, it is single center study, with relatively small sample size and small number of events. The selection of the cement was based on clinical decision with no standard criteria. In addition, information about factors that may influence the risk of infection, such as, length of surgery, reason leading to the arthroplasty, medications, immunosuppressing conditions, as well as smoking were not collected. The reasons of revision arthroplasty in the group of 50 patients were not reported. Further, procedures done by different surgeons and analysis was not stratified by surgeon. Finally, although we attempted to control for potential confounding variables, we cannot rule out the possibility of residual or unmeasured ones. However, this study adds to the current debate in the literature and the first to be published in the Kingdom. The single center may serve as a potential advantage as the criteria used to elect ALBC by surgeons in multicenter studies or national registries are less consistent. Finally, about 88% of the study population underwent TKA where the data is more debatable in the literature than THA.

In conclusion, our findings demonstrated that the routine use of ALBC in primary TJA was not associated with a decrease in the infection rates compared to PC. We acknowledge however, the limitations of our study. Since the use of ALBC has notably increased worldwide, large prospective multicenter studies are warranted to evaluate its impact on the rates of PJI and to estimate the adverse effects associated with its routine use.

## Ethics approval and consent to participate

This research followed the relevant guidelines and regulations of the Helsinki Declaration. IRB Approval of Research Project No. E-20–4921 was granted at the King Saud University Medical City. The need for informed consent was waived by the Human Research Ethics Committee.

## Authors' contributions

AMA contributed to the idea generation, editing, and literature review, TAA contributed to the methodology, data analysis, led writing, editing, and literature review, IA contributed to the literature review and data collection. WA, MA, YSA, and NHA participated in inception, data collection, writing, editing, and literature review. All authors read and approved the final manuscript.

## Declaration of Competing Interest

The authors declare that they have no known competing financial interests or personal relationships that could have appeared to influence the work reported in this paper.
